# Identification of ecdysteroids and ecdysteroidogenic genes in dragonflies and damselflies

**DOI:** 10.1038/s41598-025-08387-3

**Published:** 2025-07-01

**Authors:** Genta Okude, Mari H. Ogihara, Minoru Moriyama, Takahiro Yamagishi, Hiroshi Yamamoto, Takema Fukatsu, Ryo Futahashi

**Affiliations:** 1 Department of Biological Sciences, Graduate School of Science, The University of Tokyo, Tokyo, 113-0033 Japan; 2 Molecular Biosystems Research Institute, National Institute of Advanced Industrial Science and Technology (AIST), Tsukuba, 305-8566 Japan; 3 Institute of Livestock and Grassland Science, National Agriculture and Food Research Organization, Tsukuba, 305-0901 Japan; 4 Department of Integrated Biosciences, Graduate School of Frontier Sciences, The University of Tokyo, Kashiwa, 277-8562 Japan; 5 Health and Environmental Risk Division, National Institute for Environmental Studies (NIES), Tsukuba, 305-8506 Japan; 6 Graduate School of Life and Environmental Sciences, University of Tsukuba, Tsukuba, 305-8572 Japan; 7 Present Address: Division of Applied Biosciences, Graduate School of Agriculture, Kyoto University, Kyoto, 606-8502 Japan

**Keywords:** Insect metamorphosis, Molting hormone, Ecdysteroids, Dragonfly, Damselfly, RNA-sequencing, Evolution, Physiology

## Abstract

Ecdysteroids are critical in regulating biological processes such as ecdysis, metamorphosis, embryogenesis, and reproduction in insects. Nevertheless, the ecdysteroid repertoire and expression patterns of their synthesis genes in Odonata (dragonflies and damselflies), which belong to the most-ancestral winged insect group, have remained elusive. In this study, we examined the ecdysteroid profile of eight Odonata species and the ecdysteroid fluctuation during metamorphosis in the damselfly *Ischnura senegalensis* (Zygoptera, Coenagionidae) and the dragonfly *Pseudothemis zonata* (Anisoptera, Libellulidae). We found that ecdysone and 20-hydroxyecdysone (20E) titers corresponded to the progression of ecdysis in the penultimate nymphal instar and metamorphosis in the final nymphal instar, whereas 7-dehydrocholesterol was consistently present in the hemolymph of all the examined species and developmental stages. Considering that a higher amount of 20E was detected than ecdysone, 20E is important for inducing ecdysis and metamorphosis in Odonata, like other insects. We also confirmed that the majority of ecdysteroidogenic genes were conserved in Odonata, and their stage- and region-specific expression patterns were examined in *I. senegalensis* and *P. zonata*. Unexpectedly, most ecdysteroidogenic genes were expressed in a variety of tissues. Our study provides insights into the evolution and diversification of the ecdysteroidogenic pathway among insects.

**Supplementary Information:**

The online version contains supplementary material available at 10.1038/s41598-025-08387-3.

## Introduction

Insects, which can be regarded as one of the most successful groups of organisms, exhibit a remarkable ability to undergo metamorphosis from non-reproductive nymphs/larvae to winged adults, with a few exceptions such as primitive groups like silverfish and bristletails^1^. The process of metamorphosis in insects is orchestrated by two insect hormones: juvenile hormone (JH) and ecdysteroids^2,3^. Ecdysteroids, a group of steroid hormones, play crucial roles in insects not only in metamorphosis but also in other biological processes, such as embryogenesis, ecdysis, reproduction, and stress responses^2–4^. In most insects, 20-hydroxyecdysone (20E) is the active form of ecdysteroids^3,4^, whereas other steroids sometimes play important roles in arthropods^5^.

**Fig. 1:**
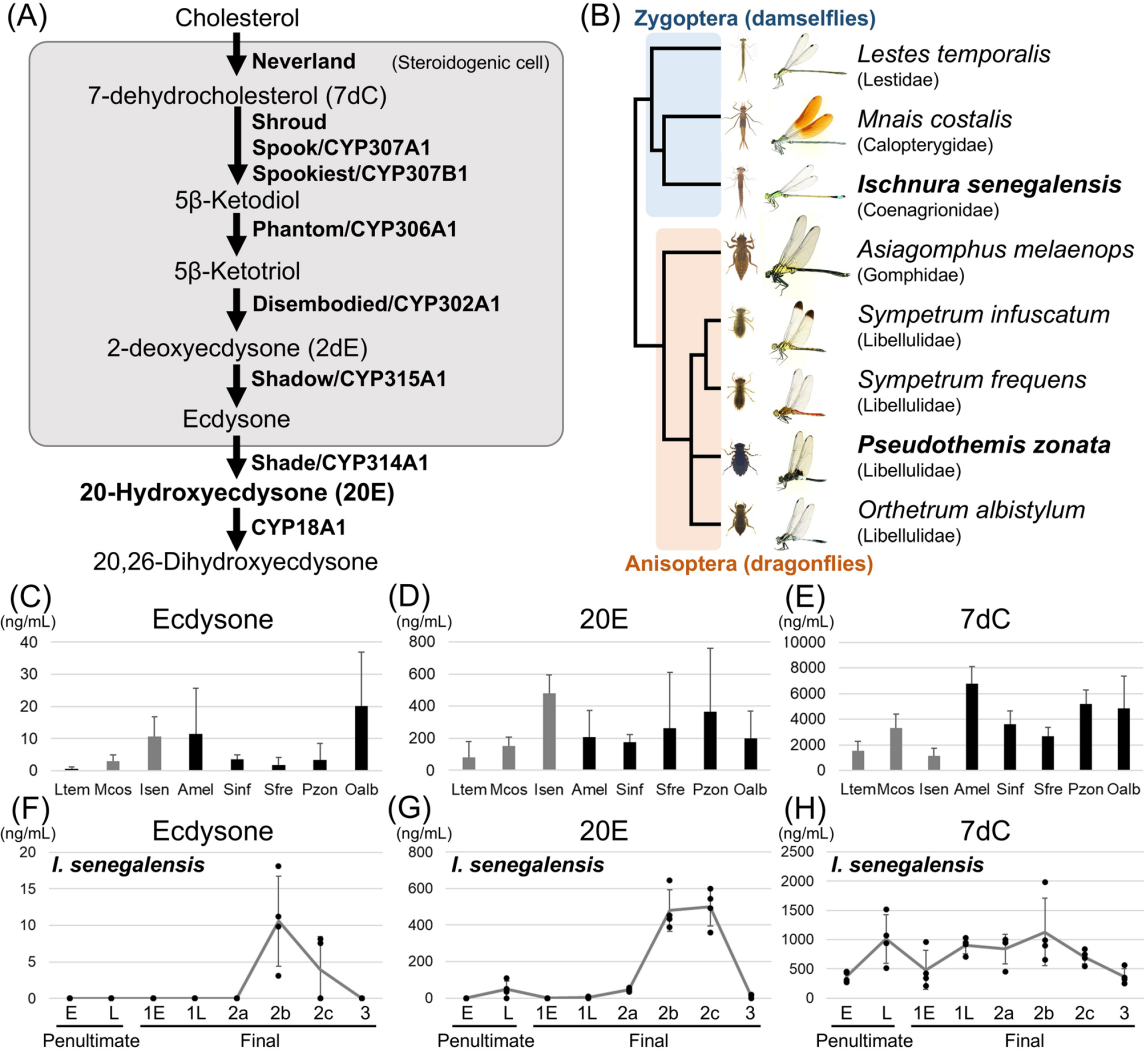
Ecdysteroids in Odonata. (**A**) Summary of ecdysteroid biosynthesis pathway in insects. (**B**) Odonata species examined in this study. Phylogenetic tree of Odonata was modified from Ozono et al.^51^. Bold species have transcriptomic data of various developmental stages. (**C-E**) Ecdysteroids detected in the hemolymph of stage 2 final instar nymphs in eight Odonata species. (**C**) Ecdysone, (**D**) 20E, (**E**) 7dC. Ltem: *Lestes temporalis*, Mcos: *Mnais costalis*, Isen: *Ischnura senegalensis* (stage 2b), Amel: *Asiagomphus melaenops*, Sinf: *Sympterum infuscatum*, Sfre: *Sympetrum frequens*, Pzon: *Pseudothemis zonata*, Oalb: *Orthetrum albystylum*. (**F-H**) Ecdysteroids detected in the hemolymph of various developmental stages in *I. senegalensis*. (**F**) Ecdysone, (**G**) 20E, (**H**) 7dC. E: Early, L: Late. Developmental stages of the final nymphal instar were based on Okude et al.^16,27^. Error bars are SD. *N* = 4 for each measurement. For visualization, values below the detection limit are displayed as zero (raw data is shown in Table S3).

The ecdysteroid biosynthesis pathway and the responsible genes have been primarily elucidated in the fruitfly *Drosophila melanogaster* and the silkworm *Bombyx mori*, which are representative holometabolous insects that undergo a pupal stage between the larval and adult stages^3,6^. As the initial step of ecdysteroid biosynthesis, cholesterol is converted to 7-dehydrocholesterol (7dC) by a Rieske oxygenase called Neverland in the prothoracic gland, which is the ecdysteroid-synthetic organ in many insects (Fig. 1A). 7dC is then converted to 5β-ketodiol through several steps known as “black box” including cytochrome P450 enzymes namely Spook (CYP307A1), Spookier (CYP307A2), Spookiest (CYP307B1), and a short-chain dehydrogenase/reductase Shroud. Subsequently, 5β-ketodiol is converted into 5β-ketotriol, 2-deoxyecdysone (2dE), and ecdysone by cytochrome P450 enzymes Phantom (CYP306A1), Disembodied (CYP302A1), and Shadow (CYP315A1), respectively. Ecdysone is released into the hemolymph and transported to peripheral tissues, such as fat body, where ecdysone is converted into 20E by a cytochrome P450 enzyme Shade (CYP314A1) (Fig. 1A). 20E is inactivated by 26-hydroxylation, which is catalyzed by a cytochrome P450 enzyme CYP18A1^7^. Most of the ecdysteroidogenic genes are conserved among both holometabolous insects^3^ and hemimetabolous insects^8^. Several ecdysteroidogenic genes are even conserved in other arthropods^9–11^.

Since the orders Odonata (dragonflies and damselflies) and Ephemeroptera (mayflies) are considered as the most-ancestral winged insects^12,13^, they are important for understanding the origin and evolution of insect metamorphosis^3,14^. We have previously described the precise developmental staging criteria for the final nymphal instars in various Odonata species^15,16^, allowing us to compare hormone titers and gene expression patterns in detail. Classical transplantation and hormone administration experiments suggest that juvenile hormone (JH) and ecdysteroids play a crucial role in metamorphosis in dragonflies, as in other insects^17–22^. In the dragonfly *Bradinopyga geminate* (Anisoptera: Libellulidae), 20E was detected in nymphs^23^, but the fluctuation in 20E titers during development remains unknown.

In this study, we identified ecdysteroids during metamorphosis in eight Odonata species (Fig. 1B) and analyzed the fluctuation of ecdysteroid titers during metamorphosis in the damselfly *Ischnura senegalensis* (Zygoptera, Coenagionidae) and the dragonfly *Pseudothemis zonata* (Anisoptera, Libellulidae) (Fig. 1). We also identified the ecdysteroidogenic genes in *I. senegalensis* and *P. zonata*, and analyzed their stage- and region-specific expression by RNA-sequencing.

## Methods

### Insect collection and rearing

For *Ischnura senegalensis*, nymphs were reared from eggs obtained from wild adult females collected in Tsukuba, Ibaraki, Japan, or from a stock strain maintained at the National Institute for Environmental Studies (NIES). A NIES stock strain (NIES-IV-8) was derived from nymphs collected by Yutaka Ogamino in NIES, Tsukuba, Ibaraki, Japan, on Jan. 31, 1993. It should be noted that all females are heterochrome in a NIES stock strain, whereas both heterochrome and androchrome females emerge from field-collected nymphs. Nymphs of the other species were collected in Tsukuba, Ibaraki, Japan. The nymphs were individually kept, and were fed with *Artemia* brine shrimp, *Tubifex* worms, or *Chironomus* midge larvae as described previously^15,16^. The developmental stage of the final instar nymphs was determined according to Okude et al.^16^.

### Ecdysteroid measurement

First, for a comprehensive identification of ecdysteroids, nymphs were thoroughly washed with tap water. The abdominal tip of each nymph was then cut using scissors and the overflowing hemolymph was collected using a glass capillary (1–5 µL or 10 µL Calibrated Pipets; Drummond Scientific). For Zygopteran species, approximately 10 µL of hemolymph from multiple individuals in the same developmental stage were pooled into the plastic 1.5 mL tube. For Anisopteran species, 10–20 µL of hemolymph was collected from a single individual. The collected hemolymph was centrifuged at 12,000 x *g* at 4 ℃ for five minutes, and the supernatants were transferred into a new tube, and a nine-fold volume of methanol was added. Each sample was then centrifuged at 3,000 x *g* at 4 ℃ for ten minutes, and the supernatant was transferred into a new tube and dried under reduced pressure. The dried samples were stored at -80 ℃ until use. Four replicates were sampled for each species and developmental stage.

Ecdysteroids in the hemolymph were quantified using liquid chromatography-tandem mass spectrometry (LC-MS/MS) as described in Zhou et al.^8^. Separation and detection of steroids were performed using a Prominence Gradient HPLC system (Shimadzu) and a triple quadrupole QTRAP 5500 mass spectrometer (AB SCIEX). Extracted steroids from the hemolymph were re-suspended with 50 µL of 100% methanol and 10 µL of samples were injected to quantification. Steroids were separated with a Pegasil ODS column (3 μm, 2.0 mm × 100 mm; Senshu Scientific Co., Ltd.) at a flow rate of 0.4 mL/min with a linear gradient of water to acetonitrile (25–99%). Chemicals using for quantification were prepared as described in Hikiba et al.^24^.

Second, to examine the fluctuations in ecdysone and 20E titers from each individual, a small hole was pierced dorsally between the head and prothorax of the nymph, and 1–2 µl of hemolymph was collected from the penultimate or the final instar nymph, respectively, using a micro-capillaries (1 µL or 2 µL; minicaps, Hirschmann). 100 µL of 50% methanol was immediately added and stored at -80 °C. Subsequently, 20 µL methanol containing 30 pg Makisterone A (Cayman chemical) was added as an internal standard. After defatting with 2 × 200 µL of hexane, the samples were mixed with 150 µL of methanol and centrifuged. The resulting supernatant was transferred to a new tube, dried in vacuo, and resuspended in 30 µL of 30% methanol. For these samples, we used another LC-MS/MS system (Acquity UPLC H-class and Xevo TQ-S micro triple quadrupole mass spectometer, Waters) equipped with an Acquity UPLC BEH C18 column (1.7 μm, 2.1 mm x 50 mm, Waters). Gradient elution with a water–methanol solution was performed at a flow rate of 0.35 mL/min.

### Phylogenetic analysis

Ecdysteroidogenic genes were searched by tblastn search^25^ against the assembled transcriptomic sequences as described in the next section. Amino acid sequences of ecdysteroidogenic genes in *Drosophila melanogaster* (annotation IDs are shown in Fig. 3) were used as a query of tblastn search. To construct the molecular phylogeny of ecdysteroidogenic genes, deduced amino-acid sequences were aligned using the Clustal W program implemented in MEGA11^26^. Molecular phylogenetic analyses were conducted by the neighbor-joining method and the maximum-likelihood method using MEGA11. Bootstrap values for neighbor-joining and the maximum-likelihood phylogenies were obtained by 1,000 resampling.

### Gene expression analysis

For gene expression analysis of ecdysteroidogenic genes, we used RNA-sequencing data of *I. senegalensis* and *P. zonata* derived from Okude et al.^27^ and additional 49 RNA-sequencing datasets. We used the entire region of the head, including areas where previous papers have shown ventral glands to be present^21^. For additional RNA-sequencing experiments in this study, total RNA was extracted from the freshly prepared samples using Maxwell 16 LEV Simply RNA Tissue kit (Promega), and complementary DNA libraries were constructed using NEBNext Ultra II Directional RNA Library Prep Kit for Illumina (NEB) and sequenced by HiSeq (Illumina). The accession numbers of RNA-sequencing data used in this study are listed in Table S1. Transcriptome analyses were conducted as previously reported in Okude et al.^27^. Adaptor and low-quality sequences were trimmed using the Trimmomatic program v. 0.36^28^, and the trimmed reads were subjected to *de novo* assembly using the Trinity program v. 2.4.0^29^. After automatic assembling, we checked and manually corrected the sequences of ecdysteroidogenic genes by using the Integrative Genomics Viewer^30^. After revising the sequences, mapping was performed using the Salmon program v. 1.5.1^31^, whereby transcript expression levels were estimated as TPM (transcripts per million) values.

## Results

### Identification of ecdysteroids in Odonata

To determine the ecdysteroids involved in the ecdysis and metamorphosis events in Odonata, we measured the levels of various ecdysteroids by LC-MS/MS in Odonata during the metamorphosis progression. Typically, in most insects, the amount of the active form of ecdysteroids (e.g. 20E) in hemolymph increases prior to the morphological changes associated with insect ecdysis and metamorphosis^3^. Thus, we measured ecdysteroids from the hemolymph at stage 2 of the final instar nymphs (during the metamorphosis progression as defined in Okude et al.^16^). Three Zygopteran and five Anisopteran species were used: *Lestes temporalis* (Lestidae), *Mnais costalis* (Calopterygidae), *Ischnura senegalensis* (Coenagionidae), *Asiagomphus melaenops* (Gomphidae), *Sympetrum infuscatum*, *Sympetrum frequens*, *Pseudothemis zonata*, and *Orthetrum albistylum* (Libellulidae) (Fig. 1B). We found that ecdysone and 20E were detected in all the species examined, with the amount of 20E being much higher than that of ecdysone (Fig. 1C, D, Table S2). Additionally, among the ecdysteroid precursors, 7dC was found to be the most abundant in all the species (Fig. 1E, Table S2). Furthermore, 2dE was detected sporadically in *S. infuscatum*, *O. albistylum*, and *A. melaenops* (Table S2). No other types of ecdysteroids (i.e. ketodiol, ponasterone A) were detected in any of the examined species.

### Quantification of ecdysteroids in *I. senegalensis*

To examine the fluctuations in ecdysteroids during ecdysis and metamorphosis, we first collected hemolymph from multiple individuals at each developmental stage of *I. senegalensis*, for which individual rearing systems have been established in the laboratory and nymphal developmental stages are well defined^15,16,27^. Hemolymph was collected from two developmental stages of the penultimate nymphal instar (early and late) and six developmental stages of the final nymphal instar (early stage 1, late stage 1, stage 2a, stage 2b, stage 2c, and stage 3). Due to the decrease of hemolymph after metamorphosis in Odonata^32^, we were unable to collect enough hemolymph from adults. Since ecdysteroid titers generally rise before ecdysis or metamorphosis in insects^3^, we focused on penultimate and final instar nymphs for ecdysteroid quantification of hemolymph in this study. As a result, the titers of ecdysone and 20E increased prior to adult emergence (i.e., stage 2 of the final instar), while the titer of ecdysone was considerably lower than that of 20E (Fig. 1F, G). The titer of 20E also increased slightly at the late stage of the penultimate instar before nymph-to-nymph ecdysis (Fig. 1G). Additionally, the titer of 7dC was constantly high at all stages examined and was not associated with metamorphosis (Fig. 1H). No other ecdysteroids (i.e. 2dE and ketodiol) were detected in any developmental stages.

**Fig. 2:**
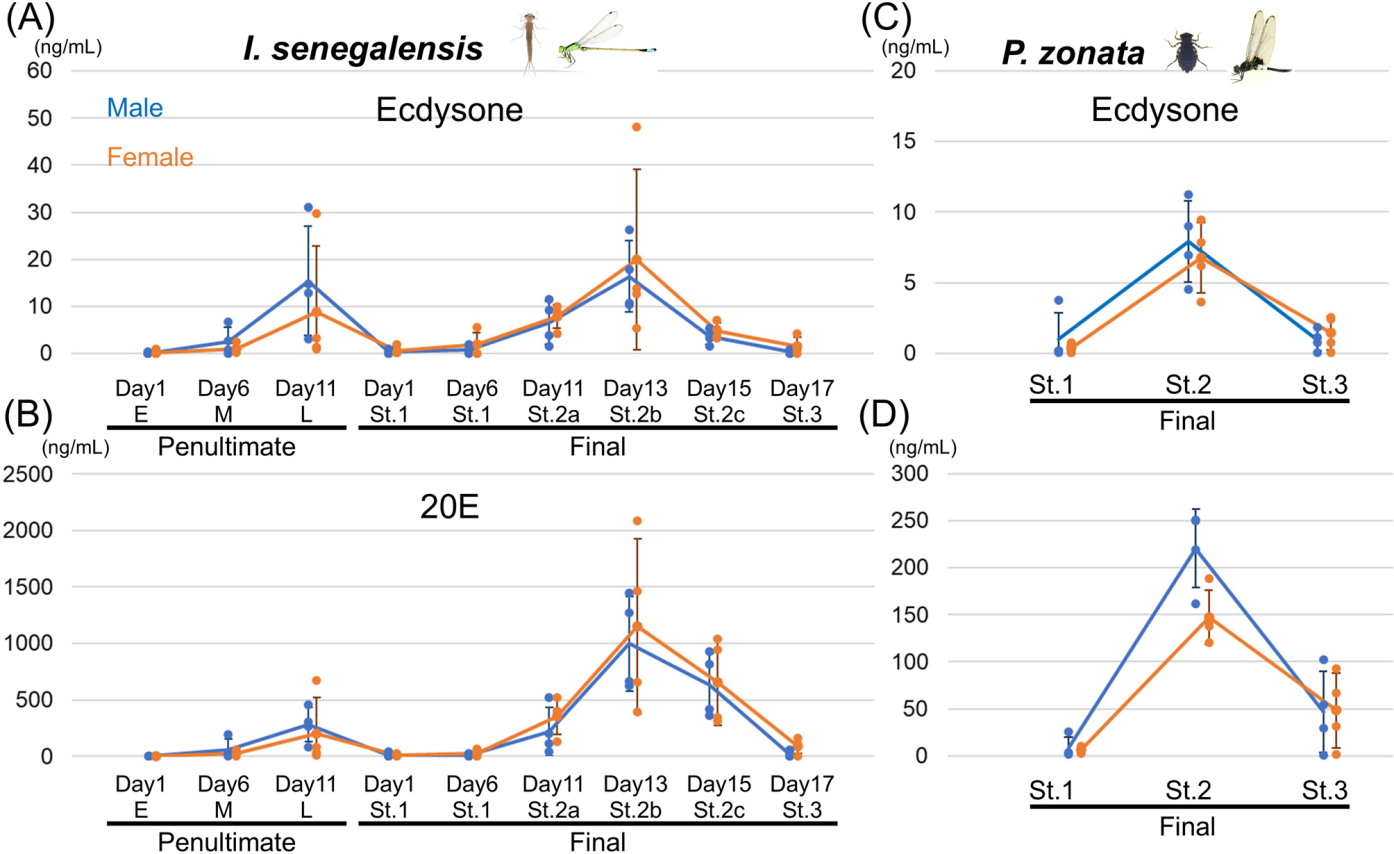
Developmental fluctuations in ecdysone and 20E titers in males and females of *I. senegalensis* and *P. zonata.* Developmental stages of the final nymphal instar were based on Okude et al.^16,27^. Error bars are SD. *N* = 4 for each measurement. For visualization, values below the detection limit are displayed as zero (raw data is shown in Table S4).

We next measured the titers of ecdysone and 20E individually by using the NIES strain of *I. senegalensis*. Like the previous pooled-samples, the titers of ecdysone and 20E increased at the late stage of the penultimate instar nymphs and at stage 2 of the final instar nymphs, associated with ecdysis and metamorphosis (Fig. 2A, B). No drastic differences were observed between sexes in the final instar nymphs, when detailed stage classification was possible, while individual differences were relatively large in the late stage of the penultimate instar nymphs, probably due to the difficulty of accurately determining the stage. We also measured the titers of ecdysone and 20E in the dragonfly *P. zonata* at three stages of the final instar nymphs and found higher levels at stage 2 (Fig. 2C, D).

### Identification of ecdysteroidogenic genes in Odonata

**Fig. 3:**
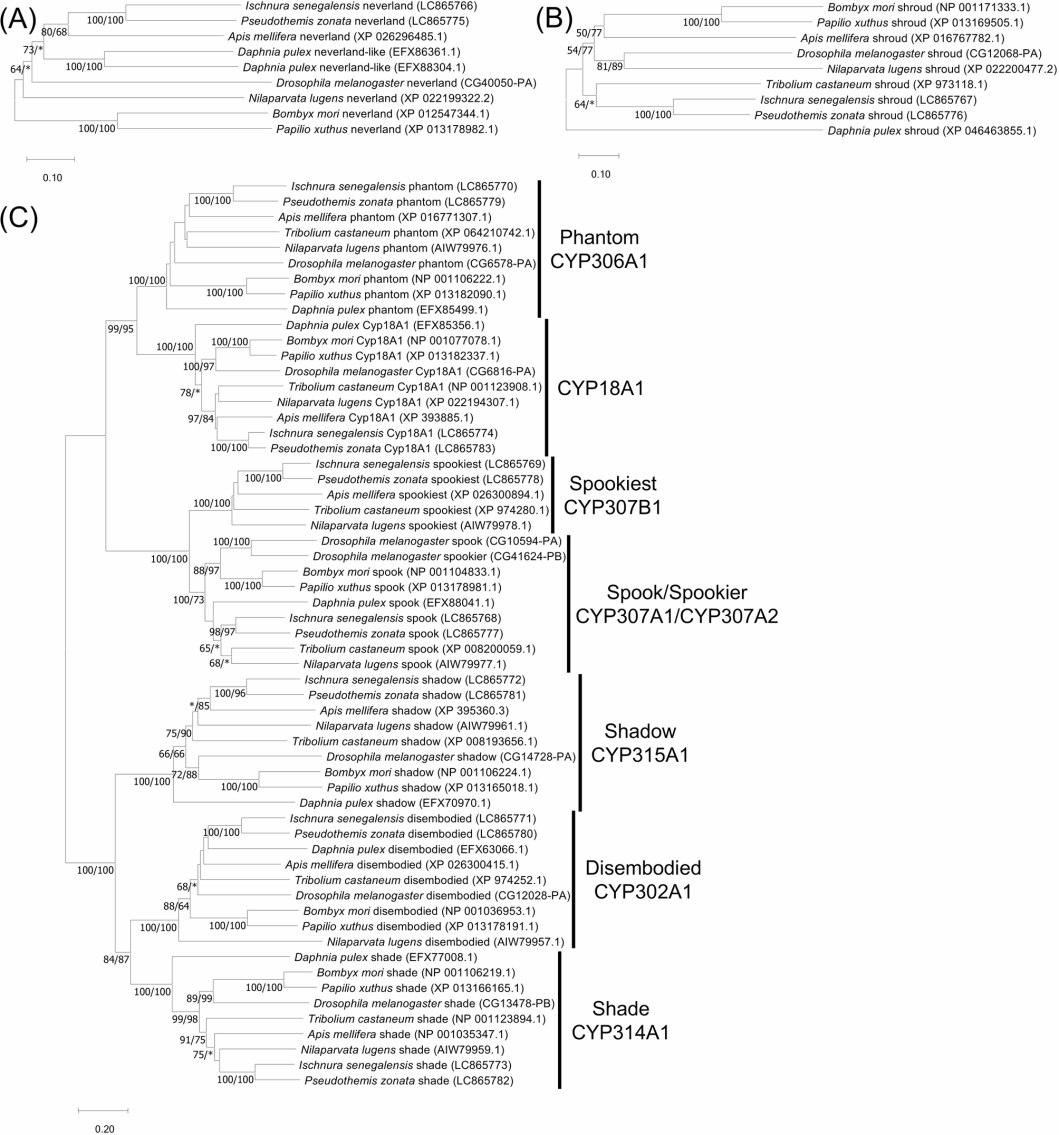
Phylogenetic tree of ecdysone synthesis genes based on their amino acid sequences. (**A**) Phylogenetic tree of Rieske oxygenase Neverland. (**B**) Phylogenetic tree of short-chain dehydrogenase/reductase Shroud. (**C**) Phylogenetic tree of P450 enzymes involved in the ecdysteroid biosynthesis. A neighbor-joining phylogeny is shown. Statistical support values for each clade are indicated in the order of bootstrap probability of the neighbor-joining analysis and bootstrap probability of the maximum-likelihood analysis from left to right, in which asterisks indicate values less than 50%. Accession numbers or annotation IDs are shown in parentheses.

We found that 7dC was abundant in the hemolymph of Odonata (Fig. 1H). Since 7dC is synthesized from cholesterol by the Neverland enzyme in steroidogenic organs such as the prothoracic gland in many insects^33^ (Fig. 1A), we next focused on the ecdysteroidogenic genes using our comprehensive transcriptome data from various developmental stages and body parts of *I. senegalensis* and *P. zonata*^27^. We identified orthologous genes of *neverland*, *shroud*, *spook/Cyp307a1*, *spookiest/Cyp307b1*, *phantom/Cyp306a1*, *disembodied/Cyp302a1*, *shadow/Cyp315a1*, and *shade/Cyp314a1*, which are well-known ecdysteroidogenic genes^6,34^, and *Cyp18a1*, which is involved in the inactivation of 20E, from the transcriptome data of both *I. senegalensis* and *P. zonata* (Fig. 3) (accession nos. LC865766– LC865783).

Like other hemimetabolous insects^34^, we could not identify the orthologous genes of *Noppera-bo*, and *spookier/Cyp307a2*, which are important for ecdysteroid synthesis in the fruitfly *Drosophila melanogaster*. We also searched for transcription factor genes, *Molting defective*, *Séance*, and *Ouija board*, which are important for transcriptional regulation of ecdysteroidogenic genes in *D. melanogaster*, but these orthologous genes were absent in the transcriptome data of Odonata as in other insects except for Diptera^34^.

### Stage- and region-specific expression of ecdysteroidogenic genes in the damselfly *Ischnura senegalensis*

**Fig. 4:**
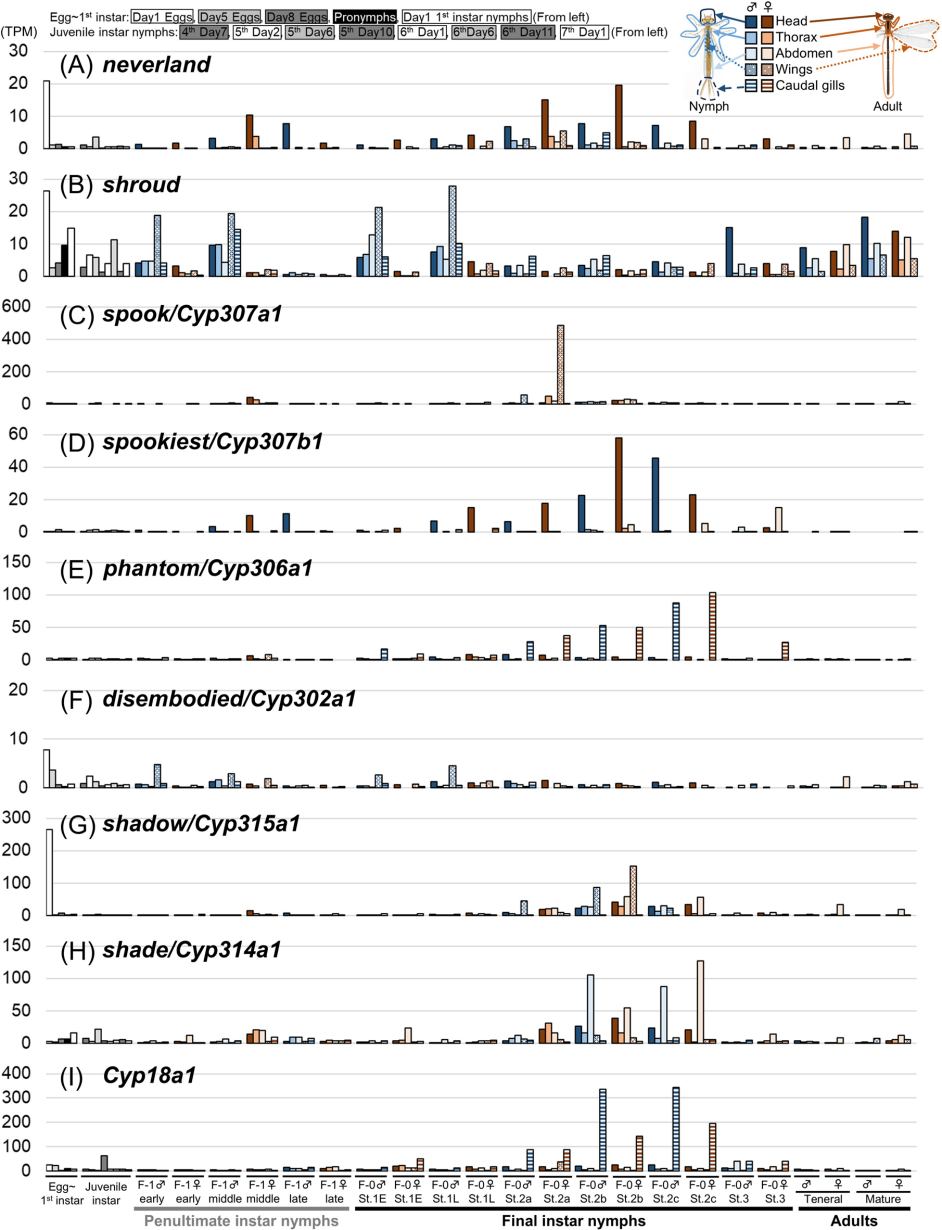
Expression patterns of ecdysteroidogenic pathway genes in the damselfly *Ischnura senegalensis*. Transcriptomic data was partly derived from Okude et al.^27^. See also Table S1.

Next, we investigated the expression patterns of nine ecdysteroidogenic pathway genes in *I. senegalensis* (Fig. 4). For the expression analysis, we added female samples of the penultimate and the final instar nymphs to our previously published dataset from various developmental stages and body regions^27^ (Table S1). In *D. melanogaster*, many of the ecdysteroidogenic enzyme genes (i.e., *neverland*, *shroud*, *spook*, *spookier*, *phantom*, *disembodied*, and *shadow*) are predominantly expressed in the prothoracic gland^33,35–37^, whereas *shade* and *Cyp18a1* are expressed in various tissues such as fat body^3,7,38^. Since classical surgical experiments suggest that the ecdysteroid-synthesizing organ (called the ventral gland in Odonata) is located ventrally to the head^21,22^, we focused on expression levels in the head region. Two genes, *neverland* and *spookiest*, were highly expressed in heads during the middle/late stages of the penultimate instar and stage 2 of the final instar nymphs (Fig. 4A, D) when the titers of ecdysone and 20E increase, as we expected. As in *Drosophil*a, *shade* was highly expressed in the abdomen during stage 2 of the final instar nymphs (Fig. 4H). Surprisingly, other genes were predominantly expressed in regions other than the head. Two genes, *spook* and *shadow*, were highly expressed in wing buds during stage 2 of the final instar nymphs (Fig. 4C, G), whereas other two genes, *phantom* and *Cyp18a1*, were highly expressed in the caudal gills, a nymph-specific organ, during stage 2 of the final instar nymphs (Fig. 4E, I). The remaining two genes, *shroud* and *disembodied* were highly expressed in the early stage of both the penultimate and final instar nymphs when titers of ecdysteroids were low (Fig. 4B, F). It should be noted that five genes, *neverland*, *shroud*, *disembodied*, *shadow*, and *shade*, were also expressed in the abdomen of adult females and during embryogenesis (Fig. 4A, B, F, G, H), although more samples are needed to consider their relevance to adult oogenesis. Similar to the results of ecdysone and 20E quantification, the expression patterns of ecdysteroidogenic genes were similar in both sexes of nymphs.

**Fig. 5:**
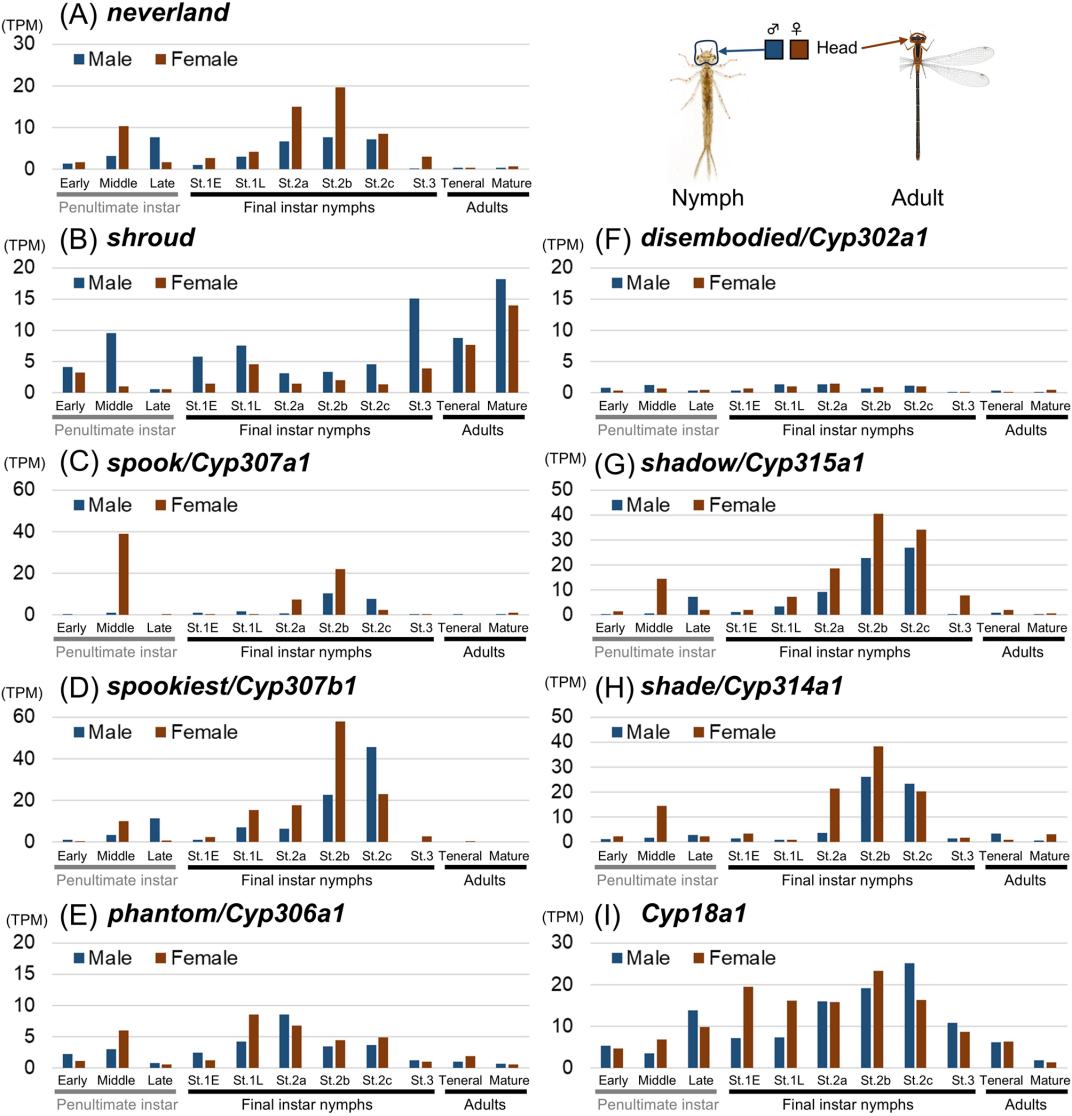
Expression patterns of ecdysteroidogenic pathway genes in the heads of the damselfly *Ischnura senegalensis*. Transcriptomic data was partly derived from Okude et al.^27^. See also Table S1.

Next, we checked the expression fluctuations of each gene in the head. In addition to *neverland* and *spookiest* (Fig. 5A, D), four genes, *spook*, *phantom*, *shadow*, and *shade*, were highly expressed in stage 2 of the final nymphal instar (Fig. 5C, E, G, H), when the titer of ecdysteroids was higher, suggesting that the ecdysteroid synthetic activity in the head contributed to the 20E production. Meanwhile, the expression of *shroud* was not correlated with the titer of ecdysteroids (Fig. 5B), and the expression level of *disembodied* was low in the head of *I. senegalensis* (Fig. 5F). The expression of *Cyp18a1*, which is involved in the inactivation of 20E, was detected in the head throughout the developmental stages (Fig. 5I).

### Expression pattern of ecdysteroidogenic genes in the dragonfly *Pseudothemis zonata*

**Fig. 6:**
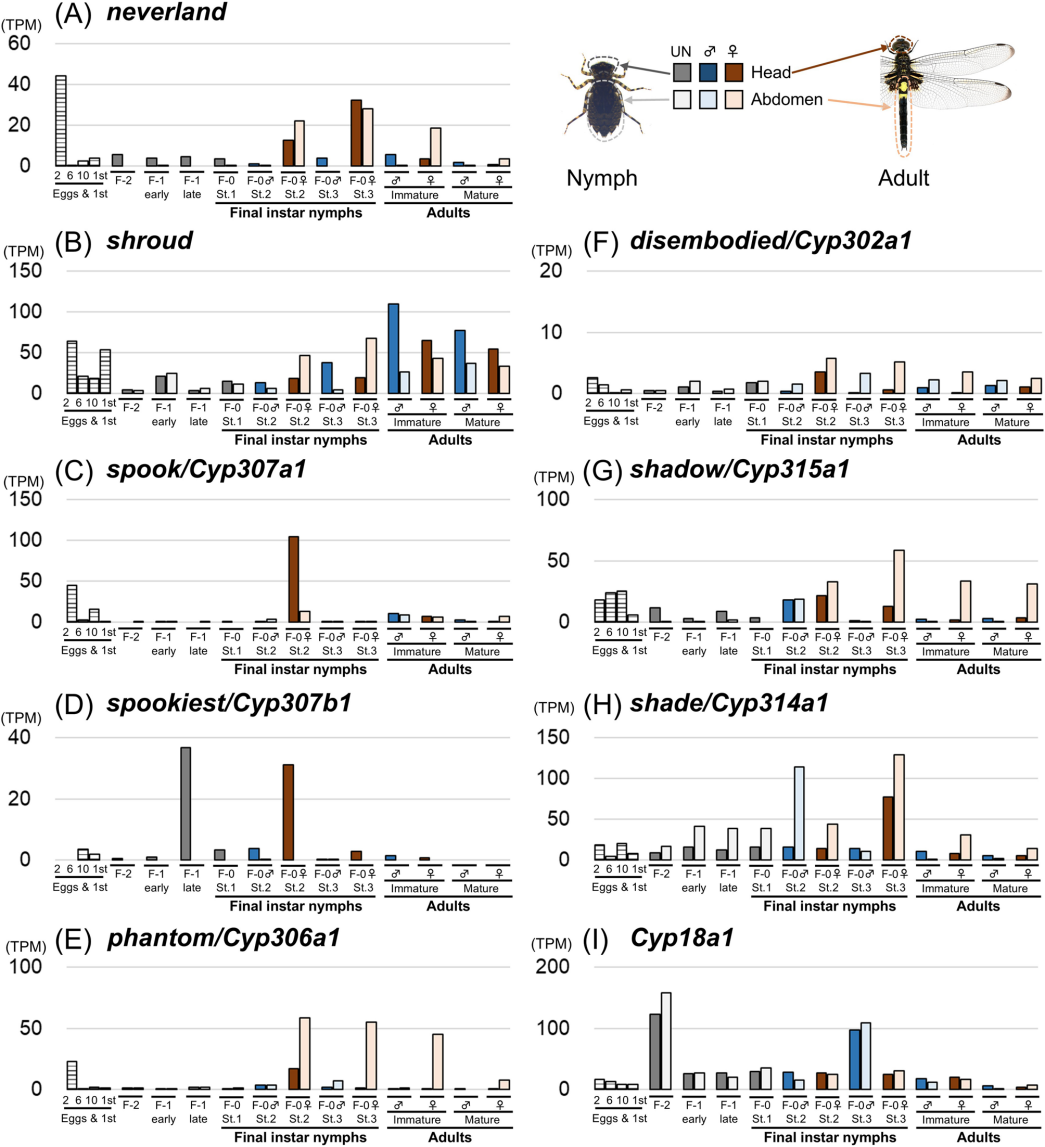
Expression patterns of ecdysteroidogenic genes in the dragonfly *Pseudothemis zonata*. F-2: Antepenultimate nymphal instar, F-1: Penultimate nymphal instar, F-0: Final nymphal instar. UN indicates individuals of unidentified sex. Transcriptomic data was partly derived from Okude et al.^27^. See also Table S1.

We also checked the expression patterns of nine ecdysteroidogenic pathway genes in *P. zonata* (Fig. 6). For the expression analysis, we added samples from eggs to the 1st instar nymphs to our previously published dataset of heads and abdomens from the antepenultimate, penultimate and final nymphal instars^27^ (Table S1). We distinguished early and late stages for the penultimate instar and three stages for the final instar using the wing morphology indicated by Okude et al.^16^. It should be noted that stage 2, which shows greater titer variation in ecdysteroids, was more unclearly distinguished in *P. zonata* compared to *I. senegalensis*, in which stage 2 can be further distinguished into three substages, 2a, 2b, and 2c^16,27^. Similar to *I. senegalensis*, *spookiest* was highly expressed in the head at late stage of the penultimate instar and stage 2 of the final instar (Fig. 6D), correlating most strongly with titer fluctuations in ecdysteroids (Fig. 2C, D). Meanwhile, the expression of *neverland* in the head remained high even at stage 3 (Fig. 6A), when the titer of ecdysteroids was reduced (Fig. 2C, D). Four genes, *spook*, *phantom*, *disembodied*, and *shadow*, showed slightly higher expression in the head at stage 2, but were also expressed in the abdomen (Fig. 6C, E, F and G), showing no clear tissue specificity. Three genes, *shroud*, *shade*, and *Cyp18a1*, showed less difference in expression among stages and regions (Fig. 6B, H and I). It should be noted that ecdysteroidogenic genes other than *spookiest* were also expressed in the abdomen of adult females and during embryogenesis (Fig. 6A, B, C, E, F, G, H).

## Discussion

### Ecdysteroids in Odonata

In all the eight Odonata species examined in this study, 7dC, 20E, and ecdysone were detected in the hemolymph during stage 2 of the final nymphal instar, and 7dC was the most abundant, followed by 20E and ecdysone (Fig. 1C, D, E). In both *I. senegalensis* and *P. zonata*, the titers of 20E and ecdysone were transiently high before ecdysis and adult emergence (Figs. 1D, G and 2), and the titer of 20E was several tens of times higher than that of its precursor, ecdysone (Figs. 1C, F and 2), suggesting that 20E acts as an active form of ecdysteroids to induce both ecdysis and metamorphosis in Odonata, as reported in other insects^3^. The increase in 20E titer at stage 2 of the final nymphal instar precisely coincides with the timing of the upregulation of *E93*, a transcription factor gene essential for adult development^27^, confirming that 20E plays a crucial role in promoting metamorphosis.

As an unexpected finding, relatively large amounts of 7dC were detected in the hemolymph of various Odonata species (Fig. 1E, H). 7dC is recognized as an intermediate in the ecdysteroidogenic pathway, which is synthesized from cholesterol by Neverland and converted to 5β-ketodiol by “black box” enzymes in the steroidogenic cells such as prothoracic gland (Fig. 1A)^6^. 7dC is generally not detected in tissues other than the prothoracic gland^39^, but the titer of 7dC is 10 times higher in hemolymph than 20E in the silkworm^40^. In *I. senegalensis*, the titer of 7dC remained high throughout the penultimate and final instar (Fig. 1H), suggesting that the 7dC titer in hemolymph was less associated with ecdysis and metamorphosis. The high levels of 7dC in the hemolymph may have important biological functions, such as serving as a precursor for vitamin D^41,42^.

### Expression patterns of ecdysteroidogenic genes in Odonata

While the fluctuation in 20E titer in hemolymph of Odonata was quite similar to that of other insects, the expression patterns of the most ecdysteroidogenic genes were less tissue- and/or stage-specific compared to those of other insects. For both *I. senegalensis* and *P. zonata*, the expression pattern of *spookiest* was most correlated with 20E titer fluctuation in hemolymph (Figs. 4D, 5D and 6D), suggesting that *spookiest* is one of the key regulators of hormone levels in Odonata. Many other ecdysteroidogenic genes were expressed at various regions (Figs. 4, 5 and 6). In hemimetabolous insects, ecdysteroidogenic genes were predominantly expressed in the prothoracic gland in the desert locust *Schistocerca gregaria*^43^ and the cockroach *Blattella germanica*^44^, while in the brown planthopper *Nilaparvata lugens*, where the prothoracic gland was not identified, ecdysteroidogenic genes were expressed in the various tissues^8^. Thus, in insects lacking a typical prothoracic gland, such as Odonata with a ventral gland, ecdysteroidogenic gene expression may not be spatially restricted to a specific tissue.

Considering the abundance of the ecdysteroid precursor 7dC in hemolymph, it is possible that ecdysteroids can be synthesized from 7dC outside of specific hormone-producing organs (e.g., prothoracic gland) in Odonata unlike other insects. Meanwhile, no intermediates of ecdysteroidogenic pathway other than 7dC were detected in the hemolymph of any developmental stages in *I. senegalensis*, although different ecdysteroidogenic genes are expressed in the various tissues of the Odonata. Intermediates other than 7dC may be rapidly metabolized or may be present only in certain tissues other than hemolymph.

Notably, *phantom* and *Cyp18a1* were highly expressed in the caudal gills during the final nymphal instar (Fig. 4E). Caudal gills are nymph-specific organs that degenerate during the final nymphal instar in damselflies^22^. Caudal gills with spread trachea are thought to be important for respiration in the water^45^, and are readily detached when the nymphs are attacked by predators^22^. Since ecdysteroids are known to regulate the degeneration of nymph-specific tissues in many insects^3^, it is possible that the upregulation of *phantom* and *Cyp18a1* in the caudal gills is involved in the degradation of this organ during metamorphosis.

In most insects, *shade* is crucial for the conversion from ecdysone to 20E and acts in the peripheral tissues^38^. In Odonata, *shade* is highly expressed in the abdomen when the 20E titer is high, suggesting that *shade* contributes to 20E production in the peripheral tissues as in other insects (Figs. 4 and 6). This expression pattern is consistent with a classical report indicating that ecdysone is converted to 20E in the fat body and Malpighian tube of the final nymphal instar in the dragonfly *Aeshna cyanea* (Aeshnidae)^46^.

In addition to ecdysis and metamorphosis, ecdysteroids have important roles in reproduction and embryogenesis^8,47,48^. In the brown planthopper *Nilaparvata lugens*, most ecdysteroidogenic genes were specifically expressed in the ovary, and gene knockdown of these genes impaired ovarian and embryonic development^8^. Both in *I. senegalensis* and *P. zonata*, several ecdysteroidogenic genes were highly expressed in the early embryonic stages and adult female abdomens (Figs. 4 and 6), suggesting the importance of ecdysteroid synthesis in ovary development and embryogenesis.

### Conclusion and perspective

In this study, we identified 20E as the primary molting hormone in both the damselfly *I. senegalensis* and the dragonfly *P. zonata*, and found that 20E titers were elevated in stage 2 of the final nymphal instar (Figs. 1 and 2), when morphological changes are most pronounced^16^. These findings will provide important basic information for studying hormone-induced morphogenesis in dragonflies and damselflies.

Moreover, we found that expression of most ecdysteroidogenic genes was detected in a variety of body regions (Figs, 4, 6) like the brown planthopper^8^, suggesting that each tissue can also partly synthesize hormones de novo. In addition, it is possible that ecdysteroidogenic genes have acquired additional functions in Odonata. Thess hypotheses need to be examined in the future by analyzing the function of each ecdysteroidogenic gene. We have recently established an electroporation-mediated RNAi method for preventing gene function locally^49^, but this method is restricted to target tissues such as the epidermis and cannot target internal tissues such as the steroidogenic organs.

Recently, a simple method of CRISPR/Cas9-based genome editing by injecting into the mother has been reported in several insects^50^. Although there have been no reports of successful genome editing in Odonata so far, if gene functional analysis in internal tissues becomes possible, it is expected to accelerate the elucidation of the molecular basis underlying hormone-induced morphogenesis in Odonata.

## Electronic supplementary material

Below is the link to the electronic supplementary material.

Supplementary Material 1

Supplementary Material 2

## Data Availability

RNA-sequencing data obtained in this study have been deposited at the DNA Data Bank of Japan (DDBJ) under DRR656590–DRR656638 Sequences of ecdysteroid-related genes identified in this study have been deposited at the DDBJ (accession nos. LC865766–LC865783).
