# Supplementary material for: Identification of ecdysteroids and ecdysteroidogenic genes in dragonflies and damselflies
Source: Sci Rep. 2025 Jul 1;15:21971. doi: 10.1038/s41598-025-08387-3 (PMC12217148; doi:10.1038/s41598-025-08387-3)
Supplement: Supplementary file 2 — Supplementary Material 2 [file 41598_2025_8387_MOESM2_ESM.docx]

**Table S1**. Sample and RNA-sequencing data used in this study.

**Table S2**. Ecdysteroids from the hemolymph at stage 2 of the final instar nymphs of eight Odonata species.

**Table S3**. Ecdysteroids from the hemolymph of various developmental stages in *I. senegalensis*.

**Table S4**. Ecdysteroids from the hemolymph of various developmental stages by individuals of *I. senegalensis* and *P. zonata*.
